# Source Apportionment and Risk Assessment of Potentially Toxic Elements Based on PCA and PMF Model in Black Soil Area of Hailun City, Northeast China

**DOI:** 10.3390/toxics12090683

**Published:** 2024-09-20

**Authors:** Zhiwei Yang, Junbo Yu, Ke Yang, Qipeng Zhang, Yangyang Chen, Shaozhong Qiao

**Affiliations:** 1 Harbin Center for Integrated Natural Resources Survey, China Geological Survey, Harbin 150086, China; yangzhiwei@mail.cgs.gov.cn (Z.Y.);; 2 Observation and Research Station of Earth Critical Zone in Black Soil, Ministry of Natural Resources, Harbin 150086, China; 3 School of Earth Sciences and Resources, China University of Geosciences, Beijing 100083, China; 4 Institute of Geophysical and Geochemical Exploration, Chinese Academy of Geological Sciences, Langfang 065000, China

**Keywords:** potentially toxic elements, PCA, PMF, source apportionment, risk assessment, black soil, Hailun City, northeast China

## Abstract

This study assessed the presence of potentially toxic elements (PTEs) in China’s northeastern black soil belt, an area with limited prior research. We collected 304 soil samples (0–20 cm) from Gonghe Town, Hailun City, and analyzed the PTE contamination degree using the single-factor pollution index and Nemerow pollution index. The results demonstrated that the mean concentrations of arsenic (As), cadmium (Cd), chromium (Cr), copper (Cu), mercury (Hg), nickel (Ni), lead (Pb), and zinc (Zn) were 11.16, 0.11, 65.29, 22.56, 0.03, 27.07, 26.09, and 66.01 mg/kg, respectively. Source apportionment was conducted via correlation analysis, principal component analysis, and positive matrix factorization, identifying four main sources: natural (33.2%), irrigation (29.5%), fuel (23.4%), and fertilizer (13.2%). The ecological risk index indicated a slight ecological risk, while the human health risk showed that non-carcinogenic risks were negligible and carcinogenic risks were acceptable. Our findings emphasize the need to prioritize controlling PTEs from fertilizer, particularly cadmium, and to a lesser extent, irrigation and fuel sources, focusing on As, Pband Hg. This research provides critical insights for policymakers aiming to manage PTE contamination in black soils.

## 1. Introduction

Soil, as a vital component of the Earth’s ecosystem, plays a crucial role in supporting life and maintaining ecological balance [1,2,3]. However, increasing anthropogenic activities have led to the accumulation of potentially toxic elements (PTEs) in the soil, posing significant threats to both the environment and human health [4,5]. The presence of PTEs in soil can be attributed to various natural and anthropogenic sources [6,7,8], including industrial emissions, agricultural practices, and atmospheric deposition. These elements, even at low concentrations, can have detrimental effects on soil fertility, crop growth [9], and the food chain, eventually impacting human health [10]. Therefore, understanding the distribution patterns, assessing the ecological and health risks, and identifying the sources of PTEs are of paramount importance for the development of effective management strategies.

The black soil region of northeast China is renowned for its fertile land, which is essential for agricultural productivity [11,12]. However, this region has not been spared from the infiltration of PTEs [13,14,15], which necessitates a comprehensive evaluation of their distribution, potential risks, and sources. In this study, we employed principal component analysis (PCA) and the Positive Matrix Factorization (PMF) model, two robust statistical techniques widely recognized in environmental research [16], to apportion the sources of PTEs in the black soil area of Hailun City. PCA is a dimensionality reduction method that simplifies the complexity of large datasets by identifying the underlying patterns, while PMF is a receptor model that quantitatively apportions the contributions of different sources to observed pollutant concentrations.

Our objectives were as follows: (1) investigate the spatial distribution pattern of PTEs, (2) evaluate their potential ecological and health risks, and (3) identify the predominant sources contributing to their presence in the soil. By integrating the findings from PCA and the PMF model, we aimed to provide a clearer understanding of the pollution characteristics and prioritize management strategies to mitigate the risks associated with PTEs in the black soil of Hailun City. The findings are anticipated to bolster the formulation of policies and steer sustainable land stewardship, thereby ensuring the enduring health of the black soil and the prosperity of the communities that depend on it.

## 2. Materials and Methods

### 2.1. Study Region

**Figure 1:**
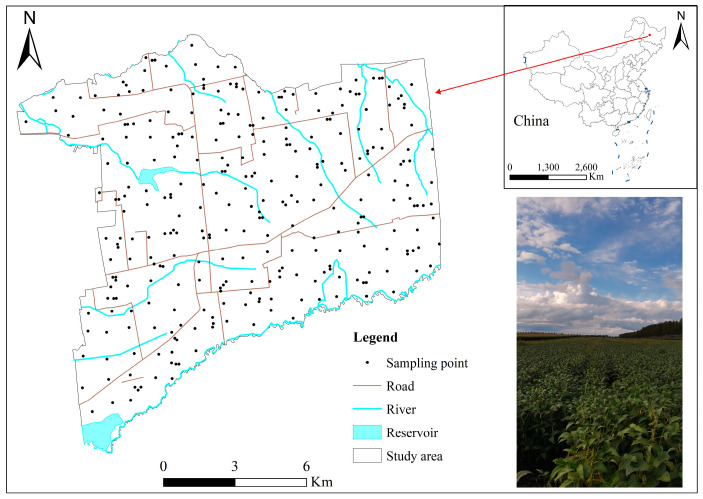
Map of study region location and sampling sites.

The study region is located in Gonghe Town, Hailun City, which is situated in the core area of black soil in northeast China, with a total area of about 174.16 km^2^, spanning the latitude 126°31′25″ to 126°45′30″ east and longitude 47°15′51″ to 47°25′16″ north, with an elevation ranging from 165 to 238 m above sea level (Figure 1). The region under investigation is defined by a continental monsoon climate, which is marked by a mean annual temperature of 2.7 °C, alongside an annual precipitation of 580.5 mm and an annual rate of evaporation that goes beyond 837 mm. The stratigraphy of the study region predominantly consists of middle Pleistocene lacustrine deposits, upper Pleistocene alluvial deposits, and Holocene floodplain deposits. The average value of the black soil layer is 65 cm thick, and its bulk density is recorded at 1.37 g/cm^−3^. Gonghe Town is primarily an agricultural community, with a focus on soybean, corn, potato, etc. In recent years, the town has witnessed the emergence of transportation, commerce, and other tertiary industries, leading to its transformation into a nascent business center [17]. 

### 2.2. Sampling and Analysis

A total of 304 surface soil samples were collected from May to August 2021, and all sampling points were located using GPS. Combined with the land use, soil type, and geological construction of the research region, the grid layout was implemented with the ArcGIS 10.8 software, with a sampling density of 8 points per km^2^. Sampling was carried out using the Plum Blossom Point method, which consists of an equal mix of five subsamples (0–20 cm) to form one sample, retaining about 1 kg of soil sample. Once collected, surface vegetation was cleared, and the samples were carefully placed in cloth bags, given unique identifiers, sealed, and then subjected to a precise weighing process. Subsequently, the samples were air-dried in a well-ventilated and clean environment, ensuring optimal conditions for preservation. Post drying, the samples underwent sieving with a 10-mesh nylon sieve to meticulously exclude plant roots, stones, and other impurities. The purified samples were subsequently divided into sample sizes and sent to the laboratory for subsequent analysis.

The analytical testing of the soil samples for PTEs and pH was conducted by the laboratory of the Harbin Center for Integrated Natural Resources Survey, which is certificated by China Metrology Accreditation. The methodologies employed for the analysis, alongside their respective detection thresholds, are shown within Table 1. All samples were pre-treated and analyzed according to NMPRGS project specifications [18]. Adhering to the precision and accuracy criteria of the national soil standards (GBW series), a random 5% subset of the samples was chosen for concurrent analysis throughout the testing process. The results demonstrated that 100% of the duplicate samples passed the test, and the quality of the data analysis was found to meet the relevant requirements.

### 2.3. Study Methods

For the purpose of this investigation, four metrics were engaged to determine the degree of soil contamination in the study region: the single-factor pollution index (*PI*), the Nemerow pollution index (*NPI*), the potential ecological risk coefficient for individual PTEs (*Er*), and the overall ecological risk index (*RI*). The grading criteria of each index are presented in Table 2 [14,19]. A quantitative description of the potential risk of soil PTEs to human health was achieved through the application of the human health risk index (*HI*). PCA and PMF were utilized to identify the origins of soil PTEs within the study region and to measure the proportional contribution of each source to establish a theoretical foundation for subsequent management strategies.

#### 2.3.1. Single-Factor Index (*PI*)

*PI* serves as a metric for evaluating the contamination level of an individual PTE within the study region [20,21]. The index can be calculated as per Equation (1):

(1) $$PI=\frac{C_{i}}{S_{i}}$$

Here, *PI* refers to the single-factor pollution index of PTEi in the soil; $C_{i}$ refers to the determined concentration of PTEi in the sample measured in mg/kg; and $S_{i}$ refers to the background value in the Songnen Plain [22]. The classification levels are presented in Table 2.

#### 2.3.2. Nemerow Pollution Index (*NPI*)

The *NPI* can be employed to evaluate the contamination status of PTEs in the soil of the study region [23]. This index can be calculated as per Equation (2):

(2) $$NPI=\sqrt{\frac{PI_{ave}^{2}+PI_{max}^{2}}{2}}$$

Here, *NPI* represents the aggregate pollution index for the sampling site; and $PI_{ave}$ and $PI_{max}$ correspond to the mean and the highest value of *PI* in the PTEs. The classification levels are presented in Table 2.

#### 2.3.3. Index of Potential Ecological Risk (*RI*)

Håkanson [21] proposed the index of potential ecological risk, which can be utilized for evaluating soil contamination by PTEs and the associated ecological risks, with the calculation detailed in Equation (3):

(3) $$RI=\sum_{i=1}^{n}E_{r}^{i}=\sum_{i=1}^{n}(T_{r}^{i}\times C_{f}^{i})=\sum_{i=1}^{n}(T_{r}^{i}\times \frac{C_{i}}{C_{n}^{i}})$$

Here, *RI* refers to the potential ecological risk index; $E_{r}^{i}$ represents the potential ecological risk index for an individual PTE; $T_{r}^{i}$ represents the corresponding toxicity coefficient for an individual PTE, with arsenic (As), cadmium (Cd), chromium (Cr), copper (Cu), mercury (Hg), nickel (Ni), lead (Pb), and zinc (Zn) having coefficients of 10, 30, 2, 5, 40, 5, 5, and 1, respectively [21]; $C_{f}^{i}$ is the contamination index for an individual PTE; and $C_{i}$ and $C_{n}^{i}$ refer to the background concentrations of PTEs in the soil. The classification levels are presented in Table 2.

#### 2.3.4. Human Health Risk Index (*HI*)

*HI* can quantitatively describe the human health hazards of soil PTEs. According to the EPA standard [24], PTEs may enter the human body via three primary routes—ingestion from hand-to-mouth activity, inhalation, and skin absorption—posing both carcinogenic and non-carcinogenic threats to human health [25]. In calculating the risk to humans from different pathways, it is necessary to distinguish between adults and children, given that the likelihood of children’s exposure to carcinogenic PTEs is higher than that of adults. The formula for adults is shown in Equations (4)–(6):

(4) $$ADD_{iing}=\frac{C_{i}\times IngR\times EF\times ED}{BW\times AT}\times 10^{-6}$$

(5) $${\mathrm{ADD}}_{\mathrm{iinh}}=\frac{C_{i}\times \mathrm{InhR}\times \mathrm{EF}\times \mathrm{ED}}{\mathrm{PEF}\times \mathrm{BW}\times \mathrm{AT}}$$

(6) $$ADD_{iderm}=\frac{C_{i}\times SA\times SL\times ABS\times EF\times ED}{BW\times AT}\times 10^{-6}$$

Here, $ADD_{iing}$, $ADD_{iinh}$, and $ADD_{iderm}$ represent the mean daily intake of a PTE through ingestion from hand-to-mouth activity, inhalation, and skin absorption; $C_{i}$ indicates the concentration of the PTE in milligrams per kilogram; and additional parameters are listed in Yu’s study [14].

The formula for children is shown in Equations (7)–(9):

(7) $${\mathrm{LADD}}_{\mathrm{iing}}=\frac{C_{i}\times \mathrm{EF}}{\mathrm{AT}}\times \left(\frac{{\mathrm{IngR}}_{\mathrm{child}}\times {\mathrm{ED}}_{\mathrm{child}}}{{\mathrm{BW}}_{\mathrm{child}}}+\frac{{\mathrm{IngR}}_{\mathrm{adult}}\times {\mathrm{ED}}_{\mathrm{adult}}}{{\mathrm{BW}}_{\mathrm{adult}}}\right)\times 10^{-6}$$

(8) $${\mathrm{LADD}}_{\mathrm{iinh}}=\frac{C_{i}\times \mathrm{EF}}{\mathrm{PEF}\times \mathrm{AT}}\times \left(\frac{{\mathrm{InhR}}_{\mathrm{child}}\times {\mathrm{ED}}_{\mathrm{child}}}{{\mathrm{BW}}_{\mathrm{child}}}+\frac{{\mathrm{InhR}}_{\mathrm{adult}}\times {\mathrm{ED}}_{\mathrm{adult}}}{{\mathrm{BW}}_{\mathrm{adult}}}\right)$$

(9) $$LADD_{iderm}=\frac{C_{i}\times EF\times SL\times ABS}{AT}\times \left(\frac{SA_{child}\times ED_{child}}{BW_{child}}+\frac{SA_{adult}\times ED_{adult}}{BW_{adult}}\right)\times 10^{-6}$$

The risks that are non-carcinogenic and carcinogenic are evaluated through the subsequent Equations (10) and (11):

(10) $$HI=\sum_{i=1}^{n}HQ_{i}=\sum_{i=1}^{n}\frac{ADD_{iing}+ADD_{iinh}+ADD_{iderm}}{RfD_{i}}$$

Here, *HI* represents the cumulative non-carcinogenic risk posed by all soil PTEs; $HQ_{i}$ signifies the non-carcinogenic risk index for an individual PTE; and $RfD_{i}$ denotes the reference dose of toxicity. A value of *HI* < 1 suggests an absence of notable non-carcinogenic risk, while *HI* > 1 suggests the presence of potential non-carcinogenic risk, the probability of which increases with increasing value [26].

(11) $$TCR=\sum_{i=1}^{n}CR_{i}=\sum_{i=1}^{n}\left(ADD_{iing}+ADD_{iinh}+ADD_{iderm}\right)\times SF_{i}$$

Here, *TCR* denotes the composite carcinogenic risk index for all PTEs; $CR_{i}$ is the carcinogenic risk associated with a specific PTE; and *SF* is the corresponding reference slope factor, which are detailed in Table 3 [14]. If *TCR* < 1 × 10^−6^, this suggests an insignificant carcinogenic risk; if 1 × 10^−6^ < *TCR* < 1 × 10^−4^, the risk is deemed acceptable; and if *TCR* > 1 × 10^−4^, it indicates a significant carcinogenic risk [26,27].

#### 2.3.5. Positive Matrix Factorization (PMF)

PMF is a multivariate factor analytical technique that operates on the principle of minimal iterative refinement [28]. The basic equations are as per Equations (12)–(15):

(12) $$X_{ij}=\sum_{k=1}^{p}g_{ik}f_{kj}+e_{ij}$$

Here, $X_{ij}$ denotes the concentration of element *j* in sample *i*; $g_{ik}$ signifies the contribution to sample *i* by factor *k*; $f_{kj}$ refers to the specific contribution from factor *k* to element *j*; and $e_{ij}$ represents the residual error for PTE *j* in sample *i*.

The calculation of the objective function *Q* is performed according to Equation (13):

(13) $$Q=\sum_{i=1}^{n}\sum_{j=1}^{m}{\left(\frac{x_{ij}-\sum_{k=1}^{p}g_{ik}f_{kj}}{u_{ij}}\right)}^{2}$$

Here, $u_{ij}$ is the uncertainty of PTE *j* in the sample *i*.

For cases where the concentration of an element is less than or equal to the method detection limit (MDL), the calculation of uncertainty proceeds as follows:

(14) $$u_{ij}=\frac{5}{6}\times MDL$$

For cases where the concentration of an element is more than its *MDL*, the calculation of uncertainty proceeds as follows:

(15) $$u_{ij}=\sqrt{{\left(Errorfraction+c\right)}^{2}+{\left(0.5\times MDL\right)}^{2}}$$

Here, c refers to the concentration of a single element; Errorfraction refers to the error fraction of the analytical method; and *MDL* represents the method detection limit value.

#### 2.3.6. Source-Oriented Potential Ecological Risk and Human Health Risk

By employing the PMF model to identify the contributions of each PTE to disparate pollution sources and amalgamating these data with the ecological risk assessment results, the degree to which different sources contribute to ecological risk was established [29]. The methodology for this calculation is outlined in Equations (16) and (17):

(16) $$RI_{j}=\sum F_{ij}\times RI_{i}$$

(17) $$D_{j,RI}=\frac{RI_{j}}{RI}$$

Here, $RI_{j}$ represents the potential ecological risk of the source category *i*; $F_{ij}$ represents the contribution of PTEi in the source category *j*; c refers to the potential ecological risk of PTEi; $RI_{i}$ represents the contribution of the potential ecological risk of the source category *j*; and *RI* refers to the total PTE ecological risk index.

Utilizing the contributions of individual PTEs to various pollution sources as determined by the PMF model, in conjunction with outcomes from health risk assessments, the contribution of different sources to the human health risk was derived [29]. This was calculated by Equations (18)–(21):

(18) $$HQ_{j}=\sum F_{ij}\times HQ_{i}$$

(19) $$CR_{j}=\sum F_{ij}\times CR_{i}$$

(20) $$D_{j,HQ}=\frac{HQ_{j}}{HI}$$

(21) $$D_{j,CR}=\frac{CR_{j}}{CR}$$

Here, $HQ_{j}$ represents the non-carcinogenic hazard quotient of source j, while $CR_{j}$ denotes the carcinogenic risk for the same source; $F_{ij}$ refers to the concentration of PTEi in source j; $D_{j,HQ}$ indicates the percentage contribution of the non-carcinogenic hazard quotient to source j; and $D_{j,CR}$ signifies the percentage contribution of the carcinogenic risk to source j.

### 2.4. Statistical Analysis

Descriptive statistics, correlation assessments, and PCA were conducted utilizing SPSS v22.0 (IBM, Chicago, IL, USA) and Originpro 2024 (Origin Lab, Northampton, MA, USA). The soil PTE contamination source analysis work was performed using EPA PMF v5.0 (USEPA, Washington, DC, USA) with SPSS v22.0 (IBM, Chicago, IL, USA). ArcGIS 10.8 (ESRI, Redlands, CA, USA) and OriginPro 2024 (Origin Lab, Northampton, MA, USA) were employed for mapping correlations. Excel 2019 (Microsoft Inc., Seattle, WA, USA) was used for calculating the values of *PI*, *NPI*, *RI*, and *HI*.

## 3. Results

### 3.1. Statistical Characteristics and Spacial Distribution of PTEs

Table 4 illustrates the statistical synthesis of the PTE and pH values and shows that the mean concentrations of As, Cd, Cr, Cu, Hg, Ni, Pb, and Zn were 11.16, 0.11, 65.29, 22.56, 0.03, 27.07, 26.09, and 66.01 mg/kg, respectively. In comparison, the mean concentrations of As, Cd, Cr, Cu, Ni, Pb, and Zn were found to exceed the background concentrations of the Songnen Plain by factors of 1.22, 1.49, 1.54, 1.27, 1.14, 1.29, and 1.27, respectively [22], while the concentration of Hg was 0.97 of the background value.

The soil samples from the study region were weakly acidic (pH mean of 6.39). In all the samples, the PTEs did not exceed the screening value. The exception was a single sample with a Cd concentration surpassing the risk control standard’s screening value for environmental quality in soils [30]; all the PTEs of the other samples did not exceed the screening value. The variability indices (CV) of the eight PTEs analyzed within the soil samples were ranked as follows: Cd (0.36) > Hg (0.30) > As (0.12) > Pb (0.11) > Zn (0.07) >Cu (0.06) > Cr (0.05) = Ni (0.05).

**Figure 2:**
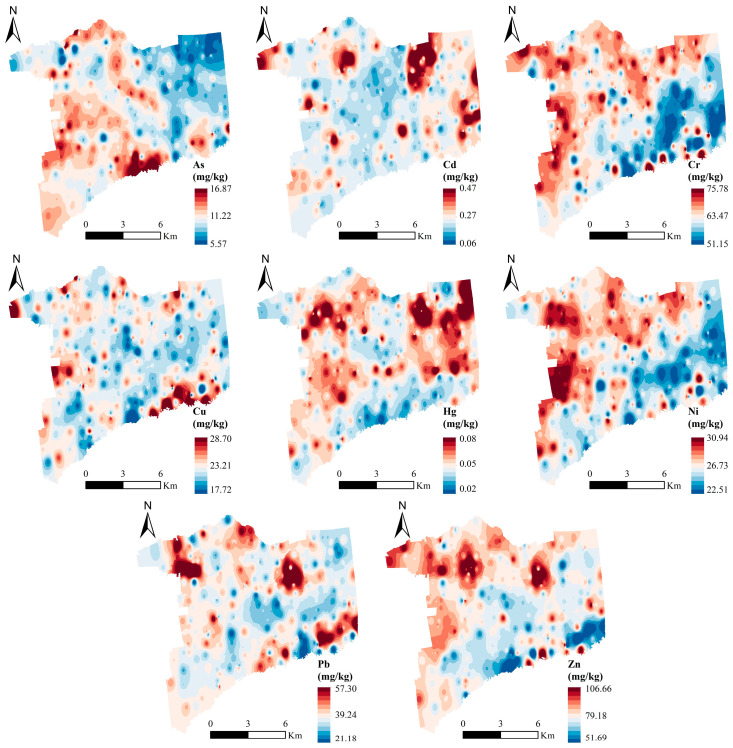
Spatial distribution of PTEs in the study region.

The spatial pattern of PTEs in the study region was obtained by the inverse distance weighting method (Figure 2). The spatial distribution of Cr, Ni, Cu, and Zn in the surface soil exhibited a similar pattern, with elevated concentrations observed in the northern and western regions, declining towards the southeast, and there were no notable peaks in the central area of the town. However, there were sporadic instances of elevated concentrations in the southeast, particularly in the vicinity of the river. The elements Cd and Hg exhibited a comparable spatial distribution, characterized by elevated concentrations near agricultural lands in rural settlements. Similarly, As and Pb showed a related distribution, with high concentrations localized to areas adjacent to rivers.

### 3.2. Status of PTEs Pollution

**Figure 3:**
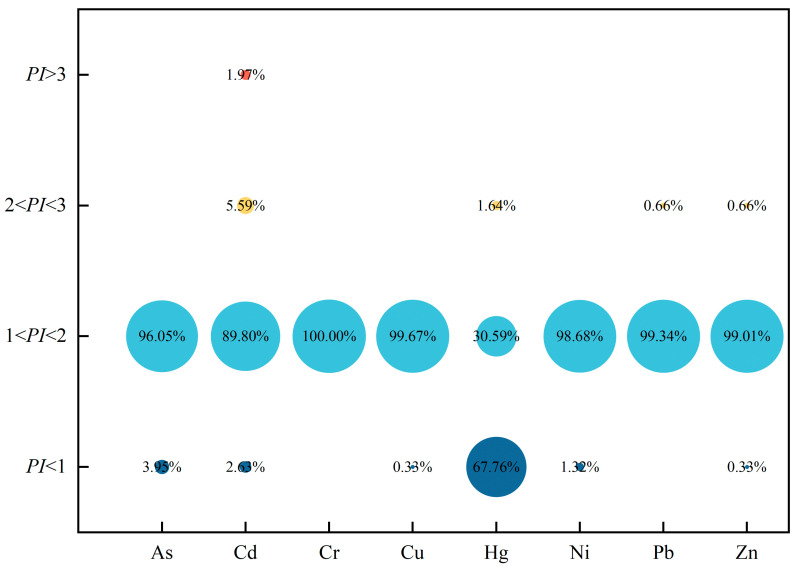
Percentage of *PI* for selected PTEs in soil at different values.

Grading based on the calculated PI values (Figure 3) showed that 3.95%, 2.63%, 0.33%, 67.76%, 1.32%, and 0.33% of the samples were As, Cd, Cu, Hg, Ni, Pb, and Zn, indicating that the samples were non-polluted (*PI* < 1). Meanwhile, 96.05%, 89.80%, 100.00%, 99.67%, 30.59%, 98.68%, 99.34%, and 99.01% of the samples were As, Cd, Hg, Pb, and Zn, indicating that they were lightly polluted (1 < *PI* < 2). In addition, Cd, Hg, Pb, and Zn moderately contaminated samples, accounting for 5.59%, 1.64%, 0.66%, and 0.66% (2 < *PI* < 3). Lastly, there were individual samples of Cd in a state of strong contamination, which accounted for only 1.97% (PI > 3).

The *NPI* outcomes showed that the majority of the soil samples (95.07%) mainly exhibited ecological risks of slight pollution (1 < *NPI* < 2). Meanwhile, 4.28% and 0.66% were classified at the thresholds of moderate pollution (2 < *NPI* < 3) and heavy pollution (*NPI* > 3). 

The results of the *PI* and *NPI* showed that there was an overall slight pollution of PTEs in the study region. Moderate and heavy PTE pollution existed in individual sampling sites, and their main polluting elements were Cd, Hg, Pb, and Zn. 

### 3.3. Potential Ecological Risks of PTEs

Table 5 presents the outcomes of the potential ecological risk index for the PTEs, with the average *Er* values arranged from lowest to highest as follows: Zn, Cr, Ni, Cu, Pb, As, Hg, and Cd. 

The *E_r_* levels associated with As, Cr, Cu, Ni, Pb, and Zn were all determined to be beneath 40, a figure that denotes a low level of ecological hazard. The *E_r_* values of Cd exhibited a range of ecological risks, with 39.47%, 56.58%, 3.29%, and 0.66% of the soil samples displaying slight, moderate, considerable, and high ecological risks, respectively. The *E_r_* values attributed to Hg revealed that 67.76% of the soil samples faced a slight ecological risk, 30.59% faced a moderate ecological risk, and 1.64% faced a substantial ecological risk. In terms of the RI, the soil samples’ values extended from 81.82 to 259.02, averaging at 118.42, which corresponded to a slight ecological risk in 93.09% of the cases and a moderate ecological risk in 6.91% of the cases. In sum, the ecological risk level of the study region was classified as slight.

### 3.4. Human Health Risk of PTEs

Table 6 displays the HQ for adults and children across three exposure pathways, listed from highest to lowest as follows: HQ for ingestion, dermal, and inhalation exposures. The HQ values for the various PTEs in the soil, ranked from greatest to least, were as follows: As, Cr, Pb, Ni, Cu, Hg, Zn, and Cd. The combined HQ values for multiple PTEs via the three exposure routes were calculated to be 1.17 × 10^−1^ for adults and 3.24 × 10^−1^ for children. The findings indicate that while children experienced a higher HI than adults, neither group surpassed the threshold of 1, which is the non-carcinogenic risk alert value, signifying no human health risk.

Given that slope factors are only accessible for As and Cd, a carcinogenic risk assessment was conducted solely for these two elements across the three exposure routes. Table 7 illustrates that the carcinogenic risks (CRs) for both adults and children, ranked from highest to lowest, were dominated by ingestion, followed by dermal and inhalation exposures. The order of carcinogenic risk for the PTEs in the population showed As being more significant than Cd. The RI for exposure to PTEs through the three pathways was calculated as 1.03 × 10^−5^ for adults and 2.60 × 10^−5^ for children. For all age groups, the carcinogenic risk fell within the acceptable range of 1 × 10^−6^ < CR < 1 × 10^−4^.

### 3.5. Source Analysis of PTEs

#### 3.5.1. Pearson’s Correlation Analysis

**Figure 4:**
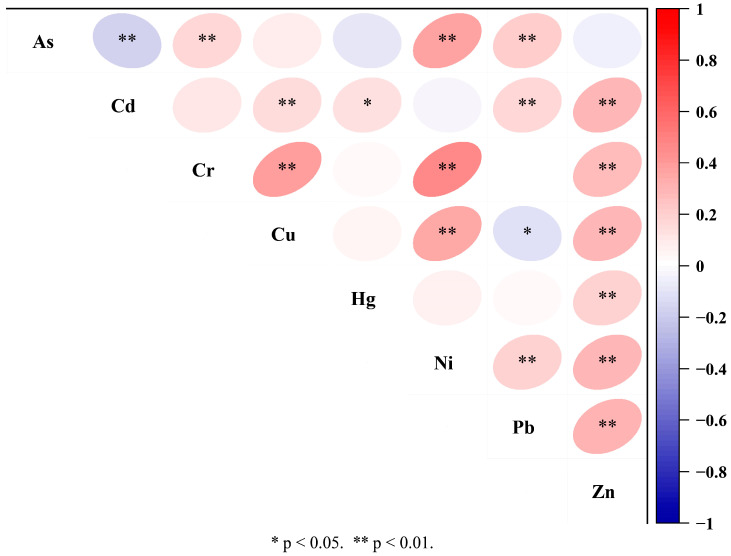
Pearson’s correlation analysis results.

Figure 4 presents the outcomes of Pearson’s correlation analysis for the eight PTEs, revealing statistically significant positive associations between Cr, Cu, Ni, and Zn (*p* < 0.01), as well as between Pb and As, Cd, Ni, and Zn (*p* < 0.01), and between Hg and Zn (*p* < 0.01).

#### 3.5.2. PCA

Prior to PCA execution, the concentration data of the PTEs successfully met the criteria of the KMO and Bartlett’s test (KMO = 0.61, Bartlett’s significance = 0.00). Table 8 displays the retention of three principal components, each with an eigenvalue exceeding 1.0, which together accounted for a cumulative variance of 61.80%. The first principal component (PC1) accounted for 27.97% of the variance, characterized by substantial positive loadings for Cr, Cu, Ni, and Zn with values of 0.708, 0.633, 0.752, and 0.653, respectively. The second principal component (PC2) represented 18.34% of the variance, with Cd and Hg showing significant positive loadings of 0.670 and 0.457, respectively. Lastly, the third principal component (PC3) elucidated 15.49% of the variance, with As and Pb exhibiting notable positive loadings of 0.358 and 0.848, respectively.

#### 3.5.3. PMF Model

**Figure 5:**
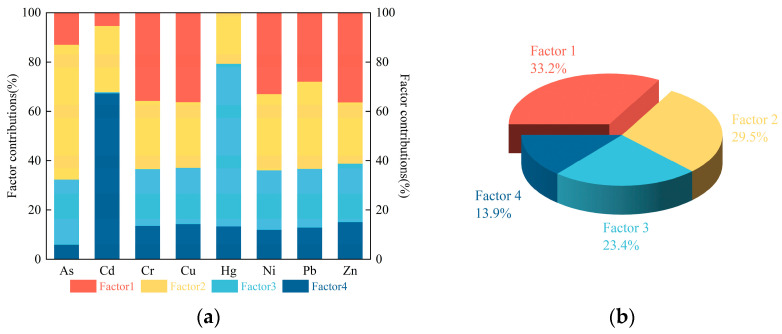
(**a**) Factor profiles of PTEs derived from the PMF model; (**b**) the percentage contribution by an individual factor using the PMF model.

The soil PTE data were subjected to source apportionment analysis using the EPA PMF 5.0 software, ensuring a signal-to-noise ratio (S/N) of at least 8.0 for each element, fulfilling the modeling prerequisites. Simultaneously, the factor count was configured to range from two to seven, with the model iterated 20 times. The determination of the most optimal factor count was achieved by evaluating the ratio of Q_robust_/Q_expected_ across various factor counts. The findings indicated that the model achieved optimal stability with a factor count of four, as illustrated in Figure 5.

Factor 1 constituted 33.2% of the overall pollution source variance, with Cr, Cu, Ni, Pb, and Zn exhibiting the most substantial loadings, contributing 35.7%, 36.2%, 33%, 30.9%, and 36.3%, respectively. Factor 2 represented 29.5% of the total pollution source variance, with As and Pb having the most significant contributions at 54.6% and 35.3%, respectively. Factor 3 made up 23.4% of the total pollution sources, with Hg being the predominant contributor at a rate of 66%. Factor 4 made up 13.9% of the total pollution sources, with Cd being the predominant contributor at a rate of 67.4%.

## 4. Discussion

### 4.1. Source Apportionment

#### 4.1.1. PCA 

PC1 primarily represented Cr, Cu, Ni, and Zn. Combined with the results of Pearson’s correlation analysis (Figure 4), there was a significant positive correlation between Cr, Cu, Ni, and Zn (*p* < 0.01), indicating that the four elements were most likely from the same source, which was consistent with the results of PCA. In terms of spatial distribution (Figure 2), the four elements of Cr, Cu, Ni, and Zn had similar spatial distribution patterns and were more evenly distributed spatially, and they are less affected by human activities and their genesis is generally related to the soil-forming parent material [31]. The geologic construction of the study region is all Quaternary stratigraphy, which is relatively homogeneous. Therefore, PC1 was described as a natural source.

PC2 primarily represented Cd and Hg. The coefficient of variation was large (>0.3), which indicates that the spatial distribution was uneven, most likely due to anthropogenic interference [32]. According to the results of *PI*, there were sporadic high values of Cd and Hg, indicating that the soil has been polluted by external factors. Combined with the spatial distribution (Figure 2), the areas of high values of Cd and Hg were located around the center of towns and villages with farmland. Long-term application of pesticides and fertilizers may lead to the accumulation of Cd in soil [30]. Hg is often released into the atmosphere in the form of vapors, which is closely related to coal combustion [16]. The study region is located in northeast China, where long and low winter temperatures require coal combustion for heating, leading to Hg accumulation and deposition [33], which are closely related to human activities. Therefore, PC2 was described as an anthropogenic pollution source.

PC3 primarily represented As and Pb. Combined with the results of Pearson’s correlation analysis (Figure 4), there was a significant positive correlation between As and Pb (*p* < 0.01), indicating that the two elements were most likely from the same source. Combined with the spatial distribution (Figure 2), the distribution areas of high As and Pb values were mainly concentrated in farmland near water facilities such as water systems, reservoirs, and irrigation canals. Zhang [34] found that that the spatial distribution of As within the soil was significantly influenced by the water system. According to fieldwork, river water is used for the irrigation of farmland in high-value areas. Meanwhile, river water polluted by industrial effluents may contain PTEs such as As and Pb, which, in turn, may contaminate farmland soil [35]. The source of As and Pb is most likely related to river irrigation. Therefore, PC3 was described as an irrigation source.

#### 4.1.2. PMF Model

Factor 1 was dominated by Cr, Cu, Ni, and Zn. The coefficients of variation for all four PTEs were less than 0.10, indicating that they are less influenced by external factors and are most likely controlled by the geologic background and soil parent material [36]. In China, Cr, Cu, and Ni are often used as indicators of natural sources [37]. Hu [38] found that Cu and Zn in soils are mainly derived from rock weathering and soil parent material. Liao [39] found that Cr, Cu, and Zn are mainly controlled by geochemistry and are derived from natural backgrounds, such as soil parent material. Huang [40] found that the enrichment of Cu and Ni in soil is likely to be influenced by the natural background. Therefore, we defined factor 1 as a natural source.

**Figure 6:**
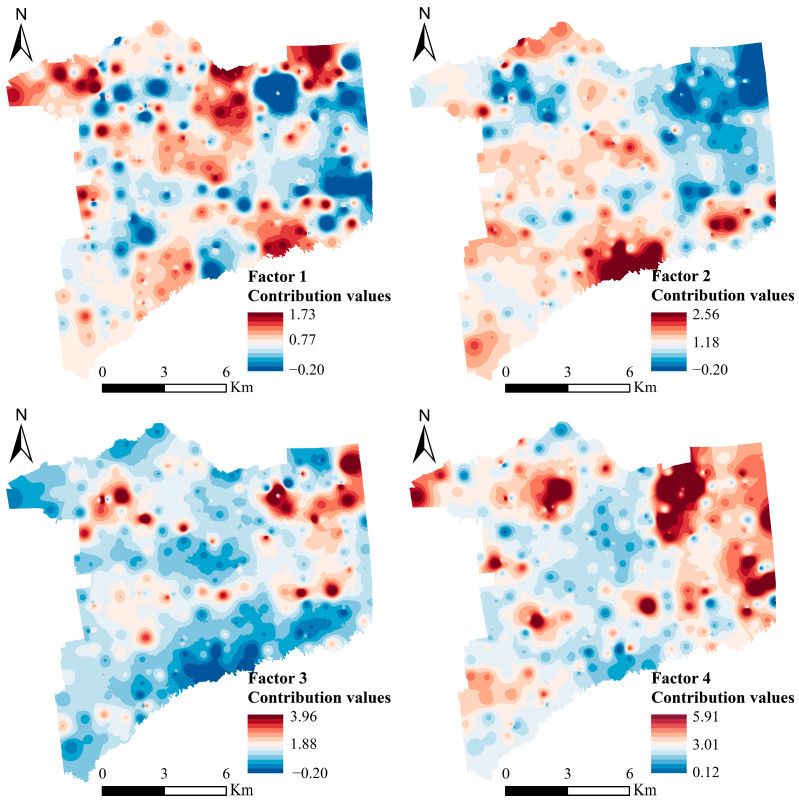
Spatial distribution of source contribution value of factors based on PMF model.

Factor 2 was dominated by As and Pb, and this factor was primarily distributed near rivers, reservoirs, and main irrigation canals in the area (Figure 6). Surface runoff carrying As-containing pollutants is injected into rivers through gullies and streams, and floodwaters overflowing the riverbanks during the flood season can pollute the soil of farmland on both sides [34]. Numerous studies have shown that wastewater irrigation can lead to an enrichment of As and Pb in soil [41,42,43]. There are factories such as brick-making factories upstream of the rivers in the study region, and there are instances of industrial wastewater discharges into the rivers. Ultimately, this leads to the accumulation of As and Pb in the soil after irrigation [44]. Therefore, we defined factor 2 as an irrigation source.

Factor 3 was dominated by Hg. By comparing the spatial distribution (Figure 2) of Hg with the distribution of the factor 3 contribution values (Figure 6), it was found that the high-value overlap area was located near the towns and villages. Hg is mainly influenced by a combination of human activities and other factors [45], and in the study area, it mainly comes from burning coal for heating in winter. The combustion of coal produces a large amount of Hg-containing vapor that is emitted into the atmosphere and subsequently deposited into the nearby soil, causing pollution [46,47]. Therefore, we defined factor 3 as a fuel source.

Factor 4 was dominated by Cd. By comparing the spatial distribution (Figure 2) of Cd with the distribution of the factor 4 contribution values (Figure 6), it was found that the areas of high concentration and contribution values overlapped. The high-value area was point-like in the farmland area, indicating that it is subject to the greatest anthropogenic influences [5]. The study region is in China’s grain production base, with a high level of agricultural mechanization and efficient fertilization techniques. It has been shown that the Cd contents of farmland are strengthened by applying fertilizers [39]. Meanwhile, due to the presence of Cd in poultry feed, the manure produced after poultry feeding is applied to farmland as fertilizer, which can lead to farmland pollution [48]. Therefore, we defined factor 4 as a fertilizer source.

#### 4.1.3. Comparison of PCA and PMF Model Source Identification

A comparison of the source apportionment results obtained from PCA and PMF revealed that they are inherently consistent. In terms of the principal component positive loadings and factor contributions, factors 3 and 4 can be regarded as further resolutions of PC2. PC1 corresponds to factor 1. PC3 corresponds to factor 2. It was found that PMF classifies the source identification results of PCA in more detail, which further validates the reliability of the source apportionment. This conclusion is consistent with the findings of Zhou [49].

In summary, comparing the results of the two methods improved the reliability and accuracy of the source apportionment. However, the source identification of the PMF is better [16], so the results of the PMF were used in the following discussions.

### 4.2. Source-Oriented Analysis of Priority Control Factors for PTEs

A source-oriented risk assessment can assist in the identification of discrepancies in the nature of hazards emanating from disparate sources [49]. To prioritize pollution sources for control, this study combined four sources of pollution with ecological and health risks.

**Figure 7:**
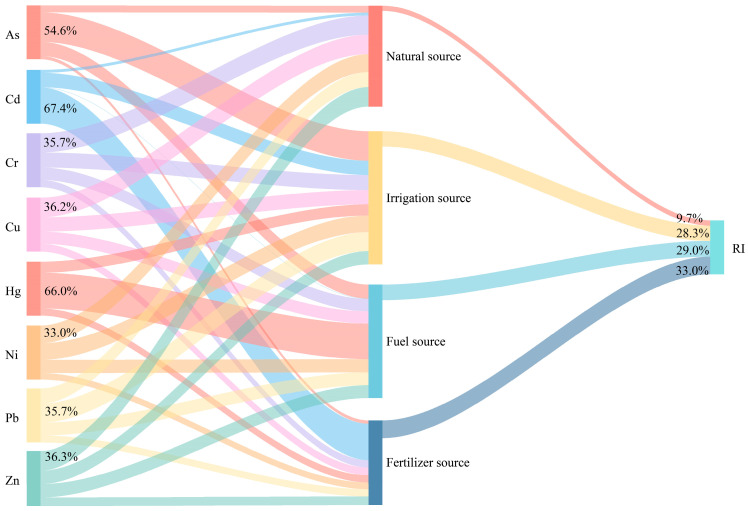
Relationship among PTEs, pollution sources, and potential ecological risk.

The pollution sources affecting the potential ecological risk of the soil were ranked in the following descending order: fertilizer source > fuel source > irrigation source > natural source (Figure 7).

The fertilizer source was the primary source of pollution affecting the *RI* of the soil, with a contribution rate of 33.0%, and the PTEs were dominated by Cd. Cadmium in soil is easily absorbed by plant roots and transferred to various organs of plants, and it is toxic to plants after accumulating to a certain concentration [50]. Some studies have shown that the consumption of Cd-containing agricultural products leads to it being more readily absorbed by the human body, far exceeding the effects on human health of direct ingestion via the three exposure routes of hand-to-mouth activity, inhalation, and skin absorption [51]. Therefore, the control of fertilizer sources should be strengthened, such as the application of organic fertilizer with a lower toxicity instead of chemical fertilizer [52]. In addition, the irrigation and fuel sources had similar contribution rates of 28.3% and 29.0%, respectively. They should be considered as secondary sources of potential ecological risks of soil, with the PTEs being dominated by As, Pb, and Hg. Low concentrations of As, Pb, and Hg may also be toxic to plants and microorganisms [53,54]. Therefore, government departments should strengthen the control of irrigation and fuel sources, for example, by reducing the discharge of factory wastewater and choosing high-quality coal as a heating fuel [55,56].

**Figure 8:**
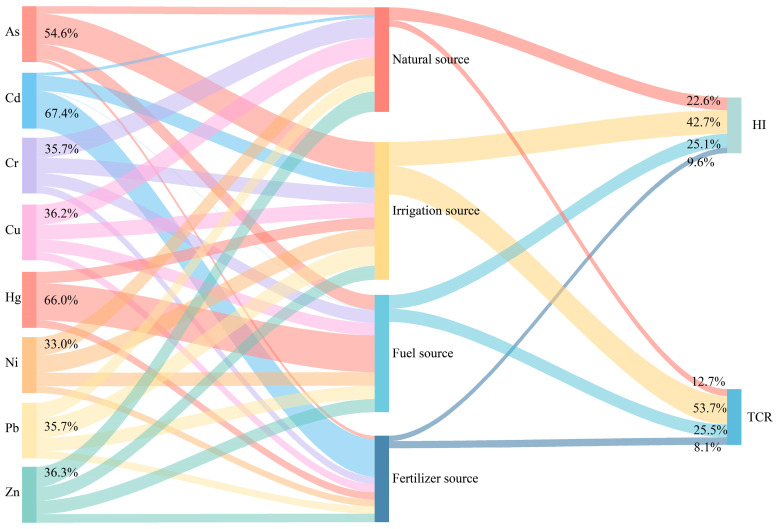
Relationship among PTEs, pollution sources, and human health risk.

The HI findings indicate that children experience elevated health risks in contrast to adults. Consequently, this study concentrated its analysis on children as a definitive group [29]. As can be seen in Figure 8, the irrigation source was the primary source of health risk, contributing 42.7% and 53.7% to the *HI* and carcinogenic risk *TCR*, respectively, with the PTEs being dominated by As and Pb. Arsenic and lead are highly toxic. Above certain concentrations, they can damage the brain and nervous system [57,58]. Therefore, control of irrigation sources needs to be strengthened. The fuel source was the secondary source of health risk, contributing 25.1% and 25.5% to the *HI* and *TCR,* respectively, with the PTEs being dominated by Hg. Mercury deposition can be enriched in the body through bioaccumulation, presenting a significant risk to human health; for instance, chronic exposure at low levels during pregnancy may lead to a decrease in the intelligence quotient of the offspring [59]. Therefore, the control of fuel sources needs to be strengthened.

The findings from the HI and RI, tailored to specific sources, exhibited discrepancies. The HI, as detailed in Table 6 and Table 7, indicated that the non-carcinogenic risks attributable to As, Pb, and Hg from irrigation and fuel sources were negligible, and the carcinogenic risks fell within acceptable limits, posing no harm. In contrast, the RI instigated by Cd from fertilizer sources was deemed moderate. Authorities should concentrate on mitigating the RI posed by PTEs, prioritizing fertilizer sources for control, with Cd as the key PTE to regulate, followed by irrigation and fuel sources, focusing on As, Pb, and Hg as secondary targets for control.

## 5. Conclusions

The study region has a slight pollution level in terms of soil PTEs. There were only individual sampling sites with moderate and heavy pollution, where the main PTEs were Cd, Hg, and Pb.

The PCA and the PMF model analyses were used to classify the PTE sources into four categories. Their respective contributions were calculated as a fertilizer source predominantly containing Cd (13.9%), an irrigation source with As and Pb as the main pollutants (29.5%), a fuel source characterized by high levels of Hg (23.4%), and a natural source with Cr, Cu, Ni, and Zn as the leading elements (33.2%).

The RI within the study region was deemed slight. However, certain sampling locations exhibited moderate ecological risk, primarily due to the presence of Cd and Hg as the predominant PTEs. For the population, including children and adults, the non-carcinogenic risk was considered insignificant, and the carcinogenic risk remained within acceptable thresholds, with As being the leading PTE of concern.

The comprehensive analysis of PTEs, pollution sources, human health ecological risks, and potential ecological risks concluded that fertilizer sources should be the primary control source and that Cd is the first PTE to control; irrigation sources and fuel sources are the secondary sources to control in the study region, and As, Pb, and Hg are the secondary PTEs to control.

## Figures and Tables

**Table 1:** Laboratory analytical methods and detection limits.

Element	Analytical Methods	Detection Limit	Digestion Method
As	AFS	0.6	Aqua regia
Cd	ICP-MS	0.01	HF + HCl + HNO_3_ + HClO_4_
Cr	XRF	2.79	Pressed powder pellets
Cu	XRF	0.85	Pressed powder pellets
Hg	AFS	0.0005	Aqua regia
Ni	XRF	1	Pressed powder pellets
Pb	XRF	1.88	Pressed powder pellets
Zn	XRF	1.5	Pressed powder pellets
pH	ISE	0.1	

The unit of PTEs is mg/kg; pH is dimensionless. See reference [14] for analytical methods.

**Table 2:** Class of indices: the single-factor pollution index (*PI*), the Nemerow pollution index (*NPI*), the potential ecological risk coefficient for individual PTEs (*Er*), and the overall ecological risk index (*RI*).

Class	*PI*	*NPI*	*Er*	*RI*
1	<1 uncontaminated	≤0.7 clean	<40 slight ecological risk	<150 slight ecological risk
2	1–2 lightly contaminated	0.7–1 warning limit	40–80 moderate ecological risk	150–300 moderate ecological risk
3	2–3 moderately contaminated	1–2 slight pollution	80–160 considerable ecological risk	300–600 high potential ecological risk
4	>3 strongly contaminated	2–3 moderate pollution	160–320 high ecological risk	≥320 significantly high ecological risk
5		>3 heavy pollution	≥320 serious ecological risk	

**Table 3:** Threshold dose and slope factors of PTEs through different pathways.

PTEs	RfD [mg/(kg∙d)]				SF [mg/(kg∙d)]		
	$ADD_{iing}$	$ADD_{iderm}$	$ADD_{iinh}$	$LADD_{iinh}$	Through Mouth	Skin	Breathing
As	3.0 × 10^−4^	3.0 × 10^−4^	3.52 × 10^−6^	5.86 × 10^−6^	1.5	1.5	4.3 × 10^−3^
Cd	1.0 × 10^−3^	2.5 × 10^−5^	2.35 × 10^−6^	3.91 × 10^−6^	6.1	6.1	6.3
Cr	3.0 × 10^−3^	7.5 × 10^−5^	2.35 × 10^−5^	3.91 × 10^−5^			
Cu	4.0 × 10^−2^	4.0 × 10^−2^					
Hg	3.0 × 10^−4^	2.1 × 10^−5^	7.04 × 10^−5^	1.17 × 10^−5^			
Ni	2.0 × 10^−2^	8.0 × 10^−4^	2.11 × 10^−5^	3.52 × 10^−5^			
Pb	3.5 × 10^−3^	5.3 × 10^−4^	8.21 × 10^−5^	1.37 × 10^−4^			
Zn	3.0 × 10^−1^	3.0 × 10^−1^					

**Table 4:** Statistics of PTEs and pH in the soil samples.

Elements	Min	Mean	Max	Median	SD	CV	Background Value	Screening Value
As	5.56	11.16	16.88	11.13	1.32	0.12	9.14	40.00
Cd	0.06	0.11	0.47	0.10	0.04	0.36	0.073	0.30
Cr	51.15	65.29	75.84	65.45	3.46	0.05	42.46	150.00
Cu	17.72	22.56	28.71	22.48	1.32	0.06	17.78	50.00
Hg	0.02	0.03	0.08	0.03	0.01	0.30	0.031	1.30
Ni	22.51	27.07	30.94	27.03	1.46	0.05	23.65	60.00
Pb	21.18	26.09	57.30	25.74	2.84	0.11	20.23	70.00
Zn	51.66	66.01	106.81	65.93	4.52	0.07	52.05	200.00
pH	5.45	6.39	8.51	6.24	0.52	0.08		

The coefficient of variation (CV); standard deviation (SD); the unit of the content of each element is mg/kg; pH is dimensionless.

**Table 5:** Potential ecological risk and proportion of heavy metals in the soil of the study region.

Parameters	As	Cd	Cr	Cu	Hg	Ni	Pb	Zn	RI
Min	6.09	23.01	2.41	4.98	19.76	4.76	5.23	0.99	81.82
Max	18.47	192.33	3.57	8.07	108.10	6.54	14.16	2.05	259.02
Mean	12.21	44.58	3.08	6.34	38.77	5.72	6.45	1.27	118.42
Class	slight	moderate	slight	slight	slight	slight	slight	slight	slight
Slight ecological risk	100%	39.47%	100%	100%	67.76%	100%	100%	100%	100%
Moderate ecological risk	0.00%	56.58%	0.00%	0.00%	30.59%	0.00%	0.00%	0.00%	0.00%
Considerable ecological risk	0.00%	3.29%	0.00%	0.00%	1.64%	0.00%	0.00%	0.00%	0.00%
High ecological risk	0.00%	0.00%	0.00%	0.00%	0.00%	0.00%	0.00%	0.00%	0.00%
Serious ecological risk	0.00%	0.00%	0.00%	0.00%	0.00%	0.00%	0.00%	0.00%	0.00%

**Table 6:** Results of non-carcinogenic risk evaluation by different exposure routes.

Element	*HQ_iing_*		*HQ_iinh_*		*HQ_iderm_*		*HI*	
	Adult	Children	Adult	Children	Adult	Children	Adult	Children
As	5.54 × 10^−2^	1.45 × 10^−1^	5.03 × 10^−4^	4.28 × 10^−4^	6.25 × 10^−3^	2.55 × 10^−2^	6.21 × 10^−2^	1.70 × 10^−1^
Cd	1.62 × 10^−4^	4.22 × 10^−4^	7.33 × 10^−6^	6.24 × 10^−6^	2.43 × 10^−5^	9.91 × 10^−5^	1.93 × 10^−4^	5.27 × 10^−4^
Cr	3.24 × 10^−2^	8.46 × 10^−2^	4.41 × 10^−4^	3.76 × 10^−4^	4.88 × 10^−3^	1.99 × 10^−2^	3.77 × 10^−2^	1.05 × 10^−1^
Cu	8.40 × 10^−4^	2.19 × 10^−3^			1.90 × 10^−4^	7.73 × 10^−4^	1.03 × 10^−3^	2.96 × 10^−3^
Hg	1.49 × 10^−4^	3.89 × 10^−4^	6.78 × 10^−8^	5.78 × 10^−7^	4.01 × 10^−4^	1.63 × 10^−3^	5.50 × 10^−4^	2.02 × 10^−3^
Ni	2.02 × 10^−3^	5.26 × 10^−3^	2.04 × 10^−4^	1.73 × 10^−4^	1.90 × 10^−4^	7.73 × 10^−4^	2.41 × 10^−3^	6.21 × 10^−3^
Pb	1.11 × 10^−2^	2.90 × 10^−2^	5.05 × 10^−5^	4.28 × 10^−5^	1.66 × 10^−3^	6.75 × 10^−3^	1.28 × 10^−2^	3.58 × 10^−2^
Zn	3.28 × 10^−4^	8.55 × 10^−4^			1.76 × 10^−5^	1.01 × 10^−4^	3.45 × 10^−4^	9.56 × 10^−4^
Total risk	1.02 × 10^−1^	2.67 × 10^−1^	1.21 × 10^−3^	1.03 × 10^−3^	1.36 × 10^−2^	5.55 × 10^−2^	1.17 × 10^−1^	3.24 × 10^−1^

**Table 7:** Results of carcinogenic risk evaluation by different exposure routes.

Element	*CR_iing_*		*CR_iinh_*		*CR_iderm_*		*TCR*	
	Adult	Children	Adult	Children	Adult	Children	Adult	Children
As	8.20 × 10^−6^	2.14 × 10^−5^	2.51 × 10^−12^	3.55 × 10^−12^	9.25 × 10^−7^	3.77 × 10^−6^	9.12 × 10^−6^	2.52 × 10^−5^
Cd	3.24 × 10^−7^	8.46 × 10^−7^	8.74 × 10^−7^	5.06 × 10^−11^	1.22 × 10^−9^	4.97 × 10^−9^	1.20 × 10^−6^	8.51 × 10^−7^
Total risk	8.52 × 10^−6^	2.22 × 10^−5^	8.74 × 10^−7^	5.41 × 10^−11^	9.27 × 10^−7^	3.78 × 10^−6^	1.03 × 10^−5^	2.60 × 10^−5^

**Table 8:** Factor loading matrix of principal component analysis.

Element	Principal Component		
	PC1	PC2	PC3
As	0.331	−0.669	**0.358**
Cd	0.289	**0.670**	0.056
Cr	**0.708**	−0.155	−0.314
Cu	**0.633**	−0.010	−0.512
Hg	0.204	**0.457**	0.001
Ni	**0.752**	−0.362	0.035
Pb	0.329	0.105	**0.848**
Zn	**0.653**	0.442	0.164
Eigenvalues	2.238	1.467	1.239
Variance contribution rate/%	27.97	18.34	15.49
Cumulative variance contribution rate/%	27.97	46.31	61.80

## Data Availability

Due to privacy restrictions, data are not available.
